# Cardiac work is related to creatine kinase energy supply in human heart failure: a cardiovascular magnetic resonance spectroscopy study

**DOI:** 10.1186/s12968-018-0491-6

**Published:** 2018-12-10

**Authors:** Refaat E. Gabr, AbdEl-Monem M. El-Sharkawy, Michael Schär, Gurusher S. Panjrath, Gary Gerstenblith, Robert G. Weiss, Paul A. Bottomley

**Affiliations:** 1 Division of MR Research, Department of Radiology, The Johns Hopkins University, Park Building, 600 N Wolfe St, Baltimore, MD 21287 USA; 2 Department of Diagnostic and Interventional Imaging, University of Texas Health Science Center at Houston, Houston, Texas USA; 3 Systems and Biomedical Engineering Department, Faculty of Engineering, Cairo University, Giza, Egypt; 4 Division of Cardiology, Department of Medicine, The Johns Hopkins University, Baltimore, MD USA; 5 The GW Heart and Vascular Institute, George Washington University School of Medicine and Health Sciences, Washington DC, USA

**Keywords:** Translational studies, Cardiac metabolism, Cardiac work, Heart failure, Magnetic resonance

## Abstract

**Background:**

It has been hypothesized that the supply of chemical energy may be insufficient to fuel normal mechanical pump function in heart failure (HF). The creatine kinase (CK) reaction serves as the heart’s primary energy reserve, and the supply of adenosine triphosphate (ATP flux) it provides is reduced in human HF. However, the relationship between the CK energy supply and the mechanical energy expended has never been quantified in the human heart. This study tests whether reduced CK energy supply is associated with reduced mechanical work in HF patients.

**Methods:**

Cardiac mechanical work and CK flux in W/kg, and mechanical efficiency were measured noninvasively at rest using cardiac pressure-volume loops, magnetic resonance imaging and phosphorus spectroscopy in 14 healthy subjects and 27 patients with mild-to-moderate HF.

**Results:**

In HF, the resting CK flux (126 ± 46 vs. 179 ± 50 W/kg, *p <* 0.002), the average (6.8 ± 3.1 vs. 10.1 ± 1.5 W/kg, *p*  <0.001) and the peak (32 ± 14 vs. 48 ± 8 W/kg, *p* < 0.001) cardiac mechanical work-rates, as well as the cardiac mechanical efficiency (53% ± 16 vs. 79% ± 3, *p* < 0.001), were all reduced by a third compared to healthy subjects. In addition, cardiac CK flux correlated with the resting peak and average mechanical power (*p* < 0.01), and with mechanical efficiency (*p =* 0.002).

**Conclusion:**

These first noninvasive findings showing that cardiac mechanical work and efficiency in mild-to-moderate human HF decrease proportionately with CK ATP energy supply, are consistent with the energy deprivation hypothesis of HF. CK energy supply exceeds mechanical work at rest but lies within a range that may be limiting with moderate activity, and thus presents a promising target for HF treatment.

**Trial registration:**

ClinicalTrials.gov Identifier: NCT00181259.

**Electronic supplementary material:**

The online version of this article (10.1186/s12968-018-0491-6) contains supplementary material, which is available to authorized users.

## Background

Heart failure (HF) is a prevalent condition with a high mortality rate that often presents with symptoms of exercise intolerance, breathlessness, fatigue, and fluid retention due to cardiac pump dysfunction [1, 2]. Energy metabolism fuels contractile function in the normal heart and impaired energy metabolism has long been hypothesized as playing a central role in HF [3, 4]. The chemical energy supporting cellular processes derives from cleavage of adenosine triphosphate (ATP) and progression to HF is associated with a declining capacity for myocellular ATP production [5] with the implication that the failing heart may be energy starved [3–9].

The primary myocardial energy reserve reaction for generating ATP is the creatine kinase (CK) reaction that reversibly transfers high-energy phosphate between phosphocreatine (PCr) and adenosine diphosphate (ADP). The pseudo-first-order forward reaction-rate constant for the CK reaction (*k*_*f*_) and the product of *k*_*f*_ and the PCr concentration (*k*_*f*_*.*[PCr]), hereinafter called the ‘CK flux’, measure the forward rate of ATP production via CK. During periods of ischemia, PCr is consumed to maintain ATP levels, reducing the myocardial PCr/ATP ratio [10, 11]. Moreover, the CK reaction has long been hypothesized to serve as a spatial and temporal buffer of the energy transfer between mitochondria, where ATP is created via oxidative phosphorylation (OXPHOS), and the myofibrils, where ATP fuels contraction [9, 12–15].

Phosphorus (^31^P) magnetic resonance spectroscopy (MRS) performed in clinical cardiovascular magnetic resonance imaging (CMR) scanners, is uniquely able to noninvasively measure the ratios and concentrations of endogenous cardiac high-energy phosphate metabolites, as well as CK flux in human hearts [8–11, 16–22]. In patients with HF and non-ischemic dilated cardiomyopathy or left ventricular (LV) hypertrophy (LVH), mean CK flux at rest can be reduced by up to 65% [8, 17], even when ATP levels are not significantly decreased. Importantly, these reductions in CK ATP supply are of a magnitude that could diminish energy availability during periods of peak cardiac demand, when peak ATP utilization is anticipated to be many-fold higher than the temporal-average ATP utilization [8, 17]. This would be consistent with the energy starvation hypothesis for HF [3–9], and could explain the observation that reduced cardiac CK flux is an independent predictor of adverse cardiac events and death [18]. However, the relationships between CK energy supply and the peak and average cardiac mechanical work in the healthy and failing human heart are unknown.

Here we combine noninvasive cine CMR volumetry with quantitative ^31^P MRS to measure both temporal cardiac mechanical work and CK energy supply in healthy subjects and patients with nonischemic HF who had reduced LV ejection fractions (EF). The data provide insight into whether the reduction in CK energy supplied to the failing heart could be sufficient to limit mechanical function, and hence provide a mechanistic rationale for targeting CK metabolism as a potential HF treatment [19]. The study tests the hypothesis that reduced temporal average and peak mechanical work is associated with reduced CK flux in HF patients.

## Methods

### Study subjects

**Fig. 1:**
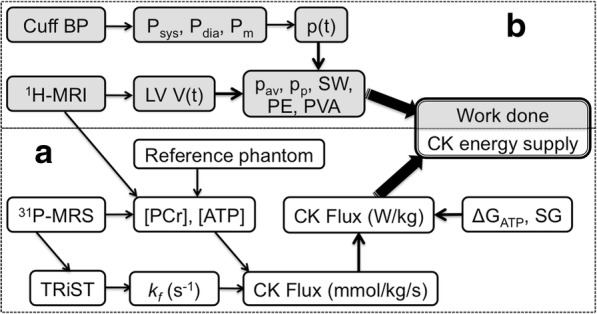
Noninvasive MRS/CMR protocol for studying cardiac energetics. **a** (bottom) ^31^P-MRS measurements of [PCr] and [ATP] concentrations in mmol/kg wet-weight using CMR volumetry and a concentration reference, is followed by triple repetition-time saturation-transfer (TRiST) measurements of the CK reaction rate, *k*_*f*_ in s^-1^, to obtain the rate of ATP production, or CK flux. The CK flux in mmol/kg/s is converted to Système International (SI) units of W/kg to compare with the cardiac workload based on the free energy of ATP hydrolysis (ΔG_ATP_) and the specific gravity (SG). **b** (top) CMR measurements of left ventricular volume, LV V(t), are combined with blood pressure (BP) cuff measures to determine the instantaneous p(t), average (p_av_) and peak (p_p_) mechanical power, and the stroke work (SW), potential energy (PE) and total mechanical energy (PVA) in Joules, Watts and W/kg (see Additional file 1). The energetics protocol takes about 50–75 min in total

We studied 27 patients over the age of 21 (age = 45 ± 14, mean ± SD yrs) with a history of HF (New York Heart Association, NYHA, class I-III) and reduced LVEF (≤45%) measured at a prior clinical imaging study using echocardiography, nuclear ventriculography, x-ray computed tomography or CMR. Subjects who had conventional contraindications to CMR, signficant valvular disease, or evidence of critical coronary disease (luminal stenosis > 50% as assessed by cardiac catheterization, computed tomography angiography, or positive stress nuclear or echocardiography) were excluded. Of the 27 patients with nonischemic cardiomyopathy, the cause was familial in 3 patients (11%) and idiopathic in 24 (89%). Fourteen age-matched healthy subjects (age = 42 ± 18 yrs) with no history of heart disease, diabetes, or hypertension served as controls. The study was performed at the Johns Hopkins University and approved by its Institutional Review Board. All participants provided written informed consent. All CMR and MRS studies were performed on a 3 Tesla scanner (Achieva, *Philips Healthcare* Best, the Netherlands) scanner equipped with 6- and 32-channel cardiac array coils and a 17-cm/8-cm ^31^P transmit/receive surface coil set [20]. The protocol for measuring CK energy supply and cardiac work is summarized in Fig. 1.

### CK metabolites and energy supply

Absolute anterior LV concentrations of [PCr] and [ATP] in mmol/kg wet weight, were determined in the anterior LV by ^31^P MRS from metabolite peak areas and an external concentration reference measured using the ‘Circle Fit’ method [23]. Concentrations were corrected for coil loading, relaxation, heart motion, tissue volume, [ATP] in ventricular blood, and coil sensitivity variations within voxels (Fig. 1a) [20]. The pseudo-first order rate-constant of the CK reaction, *k*_*f*_ in s^− 1^, was measured using the triple repetition-time saturation-transfer (TRiST) method [21] with corrections for spillover irradiation [24, 25]. The CK flux was obtained from the product, *k*_*f*_ [PCr] in mmol/kg/s, and converted to *Système International* (SI) units of Watts/kg wet-tissue weight (W/kg) by multiplying by the free-energy of ATP hydrolysis, ΔG_ATP_. The latter was taken as 60 kJ/mol, being the mean of 59–61 kJ/mol determined previously from healthy and HF patients using the same methodology [8, 19]. The MRS protocols are detailed in the Additional file 1.

### Cardiac work

**Fig. 2:**
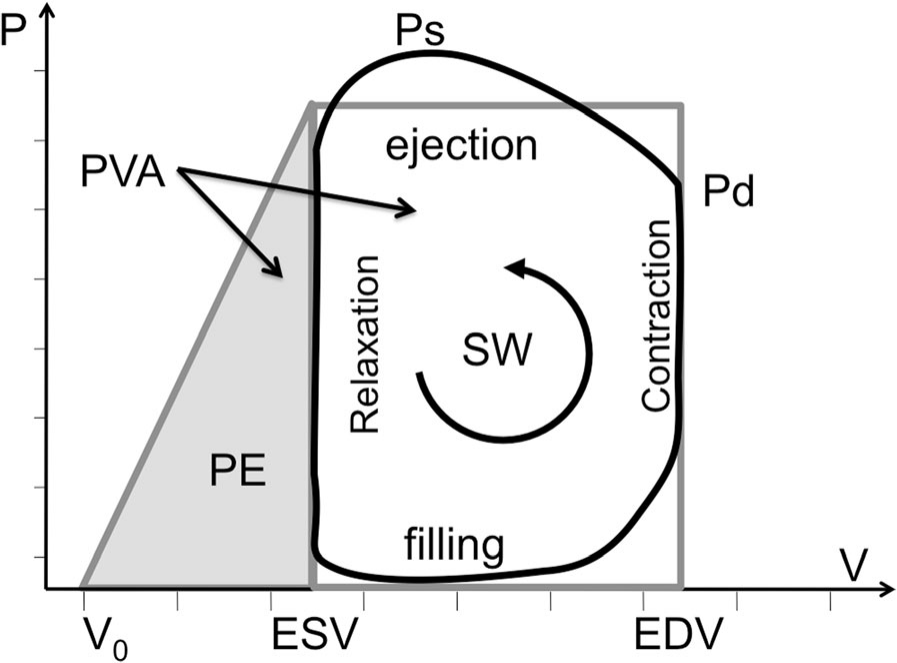
Pressure-volume schematic. The hemodynamic phases are traversed counterclockwise in one complete heart cycle (black-line). The area inside the right-hand loop defines the stroke work (SW), approximated by a rectangle (grey border); the potential energy (PE) is estimated by the end-systolic area under the grey triangle (left); and the pressure-volume area (PVA) is their sum, SW + PE. Ps and Pd are the systolic and diastolic pressures; ESV, EDV, V_0_, denote the end-systolic, end-diastolic and unstressed volumes, respectively

Most of the energy consumed by the heart is expended in ejecting blood during systole with the kinetic energy of the departing blood representing less than 1% of the pump energy (see Additional file 1). The LV whose systolic pressure and mass (LVM) are many times those of the right ventricle, performs the bulk of the cardiac work, and is in any case, the target of our MRS CK flux measurements [8, 17–22]. Cardiac work was determined noninvasively in HF patients and healthy subjects from cardiac LV pressure-volume (PV) loops (Fig. 2).

The time-dependent LV volume, *V*(t), was measured from cardiac cine CMR (Fig. 1b), using protocols also detailed in the Additional file 1. Pressure monitoring in the LV chamber requires an invasive LV catheter [26, 27] which was not feasible for these subjects. Instead, LV chamber pressure, *P*(t), was approximated by a step function scaled to the mean brachial arterial blood pressure (BP) during ejection. The PV work, *w*(*t*), was calculated from the pressure and temporal variation in LV volume, *∆V*, as:

1 $$ w(t)=-P(t)\Delta V(t) $$

The mechanical stroke work (SW) performed by the left ventricle in one cardiac cycle was measured from the area within the PV-loop which represents the integral of Eq. (1) over one cardiac cycle [28]. The instantaneous mechanical power (or rate of energy consumption) was taken as the derivative of Eq. (1), [29]:

2 $$ {p}_t=-P(t)\frac{dV(t)}{dt} $$

The internal cardiac ‘potential energy’ (PE) expended to stiffen the left ventricle prior to ejection, was estimated from the shaded region under the line connecting the end-systolic PV-point in the PV-loop (Fig. 2), to the totally unstressed volume, V_0_ at *P =* 0 [30]. As elsewhere [31], we set V_0_ = 0, a value supported by invasive studies perfomed with patients undergoing catheterization [32, 33]. The total mechanical energy (and work) was estimated by the sum, PVA = SW + PE, and the mechanical efficiency by SW/PVA [30, 34].

In addition to SW and PE, the LVM, EF and the end-diastolic (EDV), end-systolic (ESV), and stroke (SV) volumes were calculated from the MRI and BP measurements. The instantaneous mechanical power was calculated from the time-derivative of *w*(*t*) in SI units of Watts (Eq. 2). The average (p_a*v*_) and peak (p_p_) cardiac power, their ratio, and the average and peak power normalized by LVM in W/kg, were then determined and compared to the CK energy supply in W/kg.

Two-sided unpaired t-tests were used to test for statistical differences between the mechanical and metabolic measurements in healthy subjects and HF patients in accordance with the primary hypothesis, with probability *p* < 0.05 considered significant. Pearson regression analysis was used to test for correlations between CK flux and cardiac work metrics.

## Results

There were no significant differences in age, body mass index (BMI), heart-rate (HR) or systolic BP (P_sys_) between healthy and HF study subjects (Table 1), or with gender or race within groups. Among HF patients, diastolic pressure (P_dia_) was higher in African Americans than in Caucasians (82 ± 13, *n* = 14 vs. 69 ± 14 mmHg, *n* = 13; *p* = 0.05). Cardiac LVM, EDV and ESV measured by CMR were respectively 1.8, 1.7 and 3.3 times higher in HF patients than in healthy subjects (*p* ≤ 0.002).

**Table 1:**
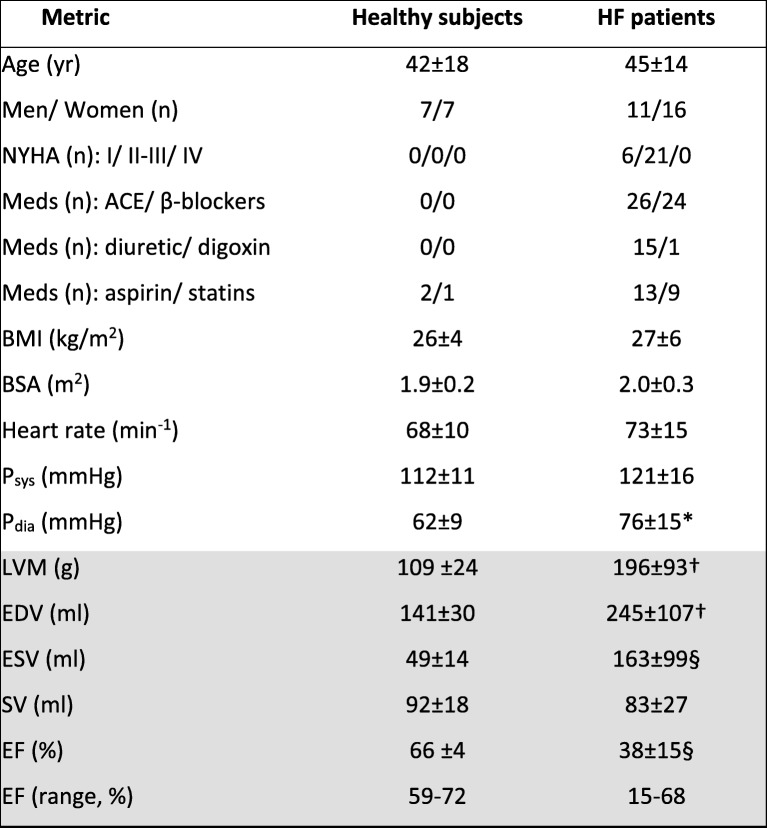
Characteristics of healthy and HF patients studied

Values are means ±standard deviation at the time of the MRI/MRS exam or numbers of subjects in each group (n). P_sys_ and P_dia_ are the resting systolic and diastolic blood pressures; *NYHA* New York Heart Association HF class, *Meds* medications, *BMI* body mass index, *BSA* body surface area. *LVM* left ventricular mass, EDV, ESV and SV are the end-diastolic, end-systolic and stroke volumes respectively; and EF = ejection fraction, measured by CMR (shaded). *p < 0.01, †*p* < 0.002 and § *p* ≤ 0.0001 vs. healthy subjects (independent 2-tailed t-test)

**Fig. 3:**
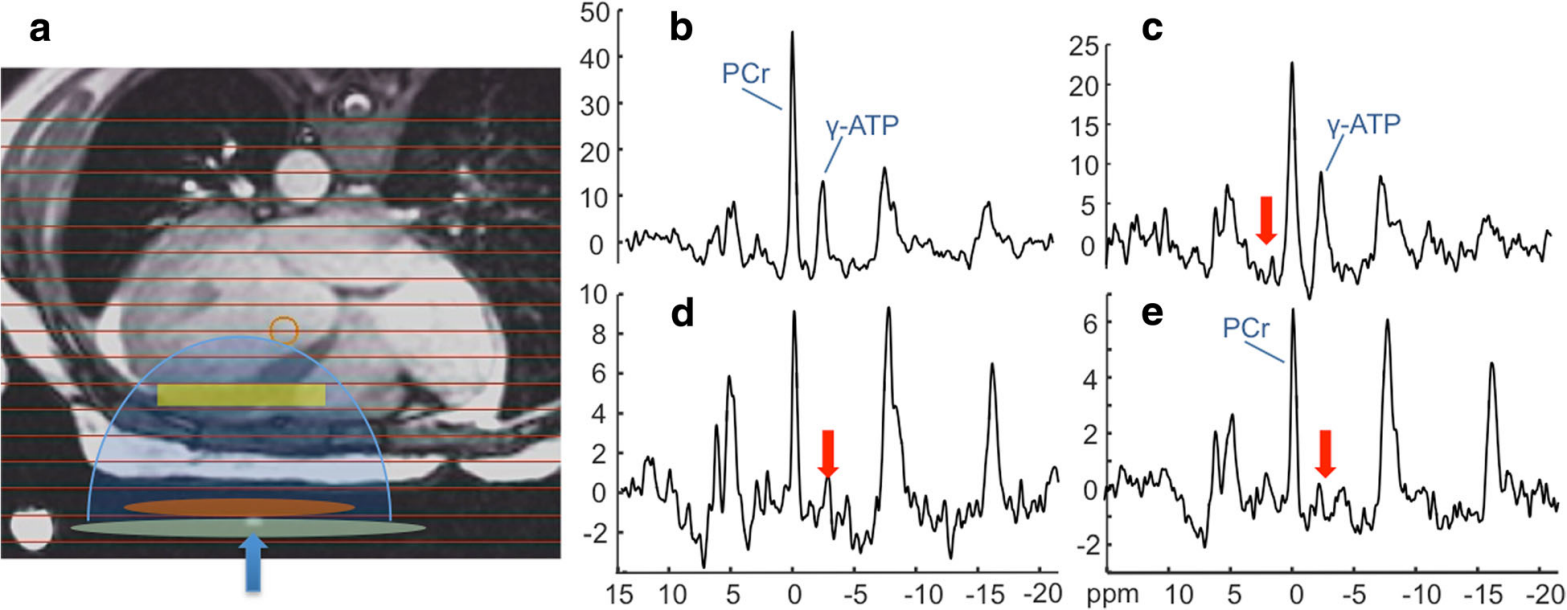
Typical ^31^P spectra from a patient with HF (age 45 yrs.; NYHA Class II). **a** Transaxial scout CMR delineating the MRS voxels (horizontal lines), and approximate locations for the transmit and receive coils (green and orange ellipses). The receiver coil’s region of sensitivity (blue shading) and an embedded coil marker (blue arrow) are also indicated. **b** Fully-relaxed spectrum from the yellow cardiac voxel in **a**, showing the PCr and ATP γ-phosphate peaks used for concentration measurements. **c**-**e** The TRiST experiment with MRS saturation (red arrows) at + 2.5 ppm as a control (**c**), and (**d**, **e**) at − 2.5 ppm to saturate the exchanging γ-ATP. The MRS repetition periods are 15.7 s, 9.7 s and 1.6 s in (c-e) respectively (see Additional file 1). The decrease in PCr in (**d**) vs. (**c**) is proportional to the CK reaction rate, *k*_*f*_ (note change in vertical intensity). The total scan time for these acquisitions was about 45 min

**Fig. 4:**
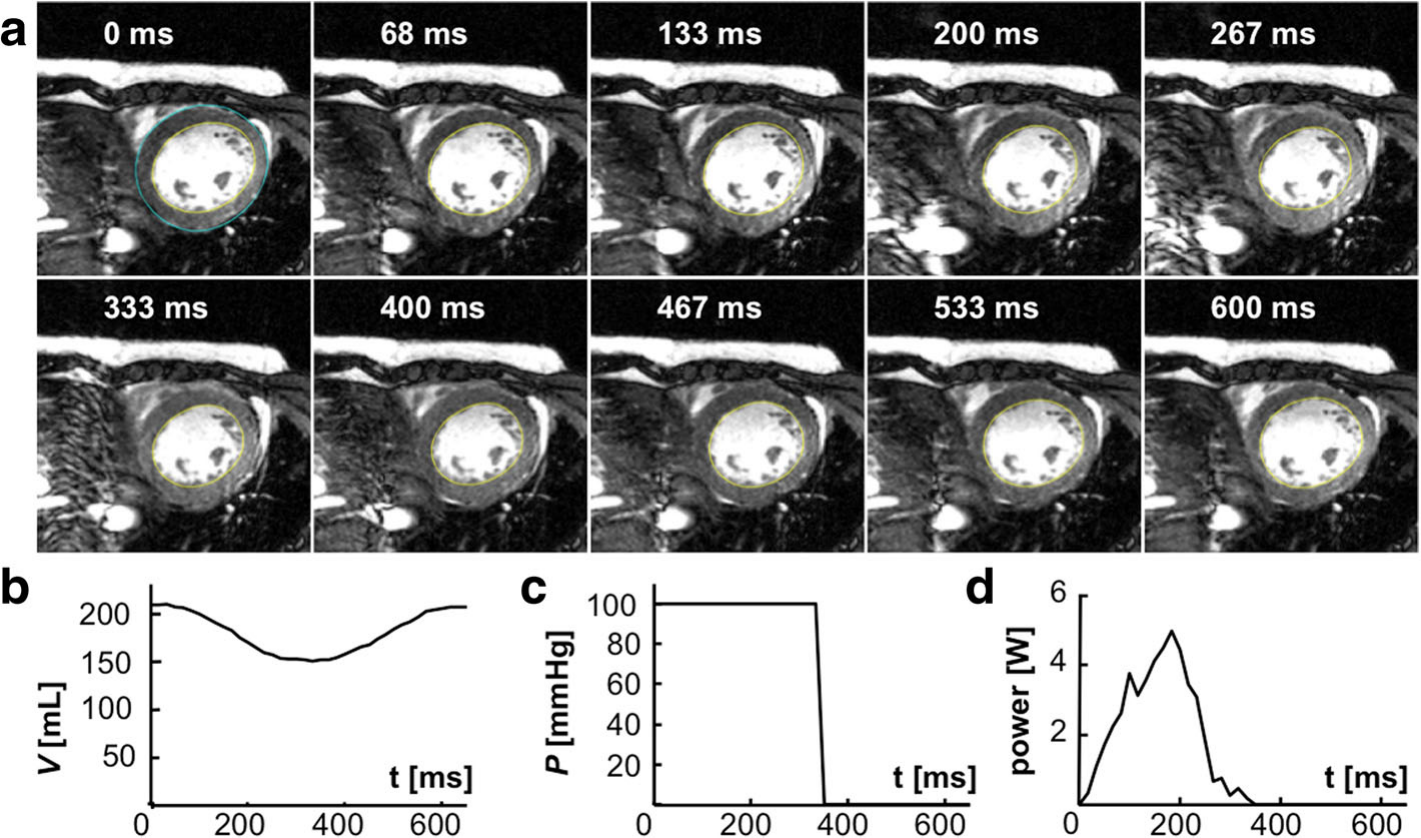
CMR and the LV volume, pressure and power waveforms for the HF patient in Fig. 3. **a** Short-axis images acquired at different times post-diastole (labeled), in a multi-slice 30-phase steady-state free-precession cine CMR (see Additional file 1). The endocardial borders used for quantifying SV are outlined in yellow (the epicardial border is blue in the first frame. **b** The volume waveform, **c** the scaled pressure model, and **d** the resulting mechanical power waveform for this patient

Figure 3 shows typical anterior LV ^31^P TRiST spectra from an LV section in a patient with NYHA class II HF. With the exchanging (γ-)ATP phosphate saturated (Fig. 3d,e) the PCr is reduced compared to the control spectrum (Fig. 2c) in proportion to *k*_*f*_ [21], which was 0.25 s^− 1^. This patient’s cine CMR, modeled pressure, LV volume and power curves are depicted in Fig. 4: the peak and average power were 4.7 W and 1.2 W, respectively.

Table 2 summarizes the mechanical and metabolic measurements from healthy subjects and HF patients. While the total SW (in Joules/beat) was preserved in HF, normalization by LVM reduced it by 33% compared to that of the healthy subjects. Potential energy, PE (Joules), was about four times higher in HF and remained double that of healthy subjects after normalization by LVM. Consequently, the total energy, PVA (Joules/beat), was 1.6-fold higher in HF patients, whereas the normalized PVA in W/kg was similar for the two groups. Because of the higher PE, mechanical efficiency (SW/PVA), was reduced by a third in HF patients (53% vs 79% in healthy subjects, *p* < 0.001). When normalized by LVM, both average and peak power were significantly reduced by 30–40% in failing compared to healthy hearts (p_a*v*_ = 6.8 W/kg vs. 10.1 W/kg; p_p_ = 32 W/kg vs. 48 W/kg, *p* < 0.0005 for both). However, because the fractional decrease in peak and average power were comparable, the peak-to-average ratio of cardiac stroke power at rest is actually the same in HF and healthy subjects, at ~ 4.8.

**Table 2:**
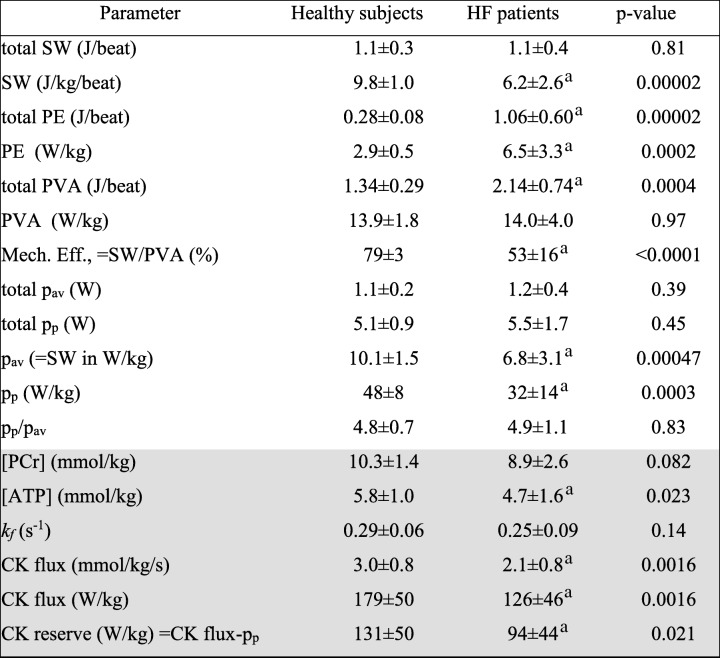
Metrics of of mechanical energy utilization and CK energy supply (shaded) measured in healthy and HF patients

Values are mean ± SD. ^a^Significantly reduced vs. healthy subjects. Abbreviations: *SW* stroke work, *PE* potential energy, *PVA* total mechanical energy; Mech. Eff. =mechanical efficiency; p_av_ and p_p_ are the average and peak stroke power, respectively; *k*_*f*_ = pseudo-first-order creatine kinase (CK) reaction-rate rate constant. [PCr] and [ATP] are the respective concentrations of myocardial phosphocreatine and adenosine triphosphate

On the supply side, the concentrations of PCr and ATP and the CK rate-constant *k*_*f*_ were all modestly reduced in HF patients by 13–19%. Consequently, the CK flux was significantly reduced by 32%, from 179 W/kg in healthy subjects to 126 W/kg in HF (*p* < 0.002). This was the same percentage reduction seen in the average and peak power in W/kg, and in mechanical efficiency. The difference between the resting CK energy supply and the normalized peak mechanical power–the CK energy reserve–was thus reduced from 131 W/kg in healthy subjects to 94 W/kg in HF (*p* = 0.02).

**Fig. 5:**
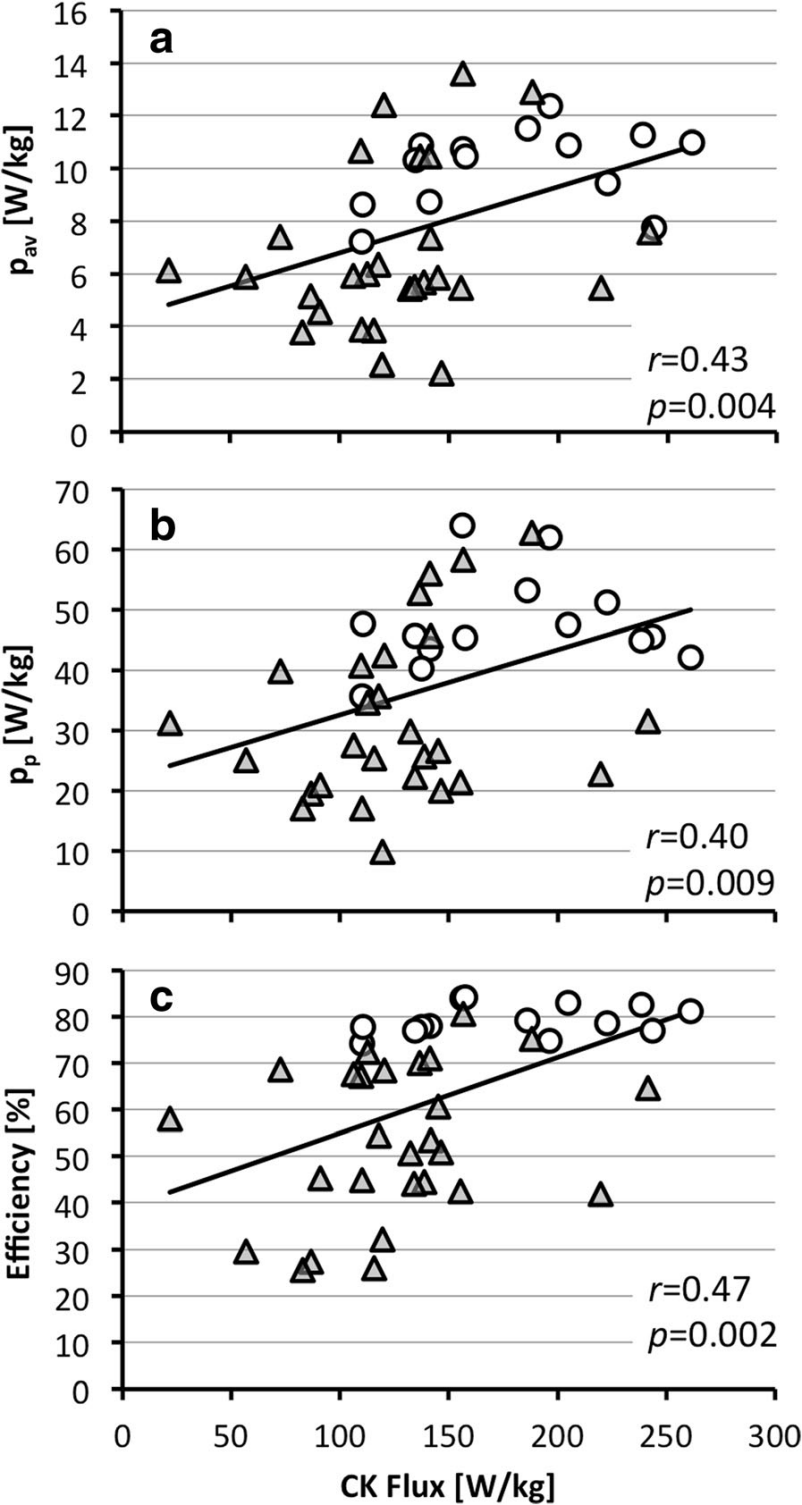
Correlation between mechanical energy and CK flux. **a** Average (p_av_) and **b** peak (p_p_) power, and **c** mechanical efficiency in healthy subjects (circles) and HF patients (triangles). Pearson correlation coefficients (*r*) and their probabilities are indicated (standard errors were 2.79, 13.1 and 16.4 respectively)

Regression analysis revealed significant correlations between the CK ATP energy supply and metrics of energy utilization (Fig. 5), namely, the average and peak power (p_a*v*_, *r* = 0.43, *p* = 0.004; p_p_, *r* = 0.40, *p* = 0.009), the PE in Joules and W/kg (*r* = 0.37, *p* < 0.02; not shown), and the mechanical efficiency (*r* = 0.47; *p* = 0.002). Among HF patients, there were no significant differences in p_a*v*_, PE, PVA, CK flux (all in W/kg) or efficiency when grouped by gender or race (*p ≥* 0.09).

## Discussion

The hypothesis that the failing heart is energy-starved and that the CK reaction–the heart’s primary energy reserve reaction–is implicated, is longstanding [3–9]. The present study is the first to noninvasively document the magnitude of the CK energy supply relative to the energy required for mechanical contraction in the same human hearts. The results have important implications for the energetics of both the healthy and failing human heart.

First, the mean basal rate of ATP production via OXPHOS previously estimated from myocardial oxygen consumption using positron emission tomography or direct sampling of coronary blood flow in non-failing human heart, is about 0.4 μmol/g wet weight/s [8, 29, 35]. This translates to about 0.4 × 60 kJ/mol =24 W/kg, which at equilibrium must equal the average rate of total energy utilization. Thus our p_a*v*_ measurement of about 10 W/kg in healthy subjects at rest would be consistent with a 10/24 ≈ 40% OXPHOS-to-SW energy conversion rate, which falls within the 25–44% range reported previously from invasive studies of animals and humans [30, 36]. In healthy subjects, our mean forward CK flux of 3.0 ± 0.8 μmol/g wet weight/s is consistent with prior ^31^P MRS measures of fluxes and rates [8, 17–19, 22, 37, 38] and translates to an average normal CK flux of 179 W/kg (Table 2). This is 179/10.1 ≈ 18 times the time-averaged SW. However, when cyclic energy demand peaks at p_p_ = 48 ± 8 W/kg (Table 2), the CK energy supply exceeds p_p_ by just a factor of 179/48 = 3.7 in healthy resting subjects.

While CK flux might also vary cyclically, there is no evidence of cyclic PCr changes in the normal heart at rest [39]. Arguably, physiological changes in *k*_*f*_ could not meaningfully register in time-frames much shorter than about 1/*k*_*f*_ ≈ 3 s anyway, and analyses of the temporal dynamics of the CK reaction and the ^31^P MRS experiment predict that cyclic variations in *k*_*f*_ would be too small to detect over physiological parameter ranges [40]. Moreover, CK flux does not change in healthy subjects when cardiac work-load is doubled [8]. If CK flux doesn’t increase with work-load, then a CK energy supply that is only 3.7-times that needed for peak cardiac SW at rest may be limiting at high work-loads in healthy subjects. Indeed, significant reductions of 14–21% in myocardial PCr/ATP have been reported at 3–4 times the resting HR × BP product in healthy subjects [41, 42], and even athletes [43].

Second, the present findings provide important support for the hypothesis that the CK reaction plays a vital role in delivering and buffering the ATP energy to fuel myofibrillar contraction in the human heart [9, 12–15], by arguably ruling out two alternative hypotheses. In order for changes in CK metabolism to have a *relevant* impact on cardiac energy supply in HF, CK flux should be neither much less than nor much more than cardiac utilization over a full-range of physiological conditions. If CK energy supply were much less than peak cardiac work, then it would be ineffectual as an energy buffer [8]. If on the other hand, the CK energy greatly exceeded cardiac work over the entire range of physiological activity, then the reductions in CK flux of 30–60% in human HF seen here and elsewhere [8, 17, 19] would not meaningfully impair energy supply. Instead, our measures of CK energy supply fall just within a range consistent with it representing a substantial source of cardiac energy, but small enough that a 30–60% shortfall could impair cardiac energetics, especially during times of stress or increased cardiac demand.

Third, cardiac CK flux *was* significantly lower at 2.1 μmol/g_ww_/s or 126 ± 46 W/kg in HF patients compared to healthy subjects (*p* < 0.002) and correlated with the work rate normalized by LVM (*p* ≤ 0.009; Fig. 5). This 31% mean reduction in CK flux is consistent with, albeit smaller than, that reported in prior HF studies [8, 17, 19]. Curiously, both average and peak stroke work rates were lower in HF by the same amount (a third) as the CK energy supply (Table 2). Consequently the ratio of the CK energy supply to the peak stroke work expended in pumping blood at rest, is 126/32 = 3.9 in HF patients–essentially the same as in healthy subjects.

While LVM increased by 1.8-fold in HF (196 vs. 109 g, *p <* 0.002; Table 1), the failing heart in total, produced the same per-beat stroke work as in healthy hearts (SW =1.1 J; Table 2). Because P_sys_ is comparable in HF and healthy subjects (Table 1), the total stroke work is essentially a measure of blood volume moved per beat (or the total change in blood volume, SV per beat). Thus the preservation of total SW (in Joule) is consistent with the preservation of SV in HF (83 vs. 92 ml, *p* = ns; Table 1) being an important early driver of cardiac remodeling [44]. Although the increase in LVM in HF is larger than the ~ 33% decline in contractile work and per-gram CK ATP energy supply, this extra mass and supply would substantially compensate for the lower mechanical efficiency of the remodeled heart (53% vs. 79% in controls, *p* < 0.001, with PE =6.5 W/kg in HF vs 2.9 W/kg in controls, *p*  <0.001). Indeed in HF, the increase in the *total* work, PVA (=SW + PE, in Joules), is comparable to the total increase in LVM (a 2.14/1.3 = 1.6-fold increase in PVA vs. 1.8-fold for LVM; *p* = ns).

Fourth, the difference between CK flux and the peak mechanical power–the CK energy reserve–nevertheless seems to provide a large surplus of energy to support cardiac function at rest in both healthy subjects (131 W/Kg; Table 2) and in HF patients (94 W/Kg). However, the reduced mechanical efficiency in HF places an additional tax on the energy available for mechanical work, if the heart is to maintain a normal peak-to-average mechanical workload (p_p_/p_av_ ~ 4.8) during stress. With a mean mechanical efficiency of 53% in HF, the CK flux should be able to support a doubling of peak mechanical power for the *average* HF patient studied here (2p_p_ /53% =121 W/kg, vs. the 126 W/kg supplied via CK flux; Table 2). But patients with more severe reductions in CK flux (e.g. 1.1–1.6 μmol/ g_ww_/s [8, 17]) or with lower efficiency (Fig. 5c), may experience more significant consequences such as worsening or activity-limiting symptoms.

### Limitations

Until the past decade, measuring cardiac mechanical work required invasive catheter-based pressure and/or volume measurements. This limited studies to patients undergoing clinical cardiac catheterization [26, 32, 33, 45–49] that generally excluded truly healthy subjects [27] and confounded comparisons with less severely compromized patients. Nevertheless, our measures of SW =1.1 ± 0.3 J/beat are not inconsistent with values of SW =0.6–1.2 J/beat [27, 31, 50] in healthy subjects and SW =0.3–1.1 J/beat [45, 48, 51] in HF (Table 3) [52]. Moreover they agree with recent noninvasive SW measurements of 1.1 J in HF and 1.2 J in healthy subjects obtained using similar CMR volumetry and arterial BP methods [31, 50, 51] as here.

**Table 3:**
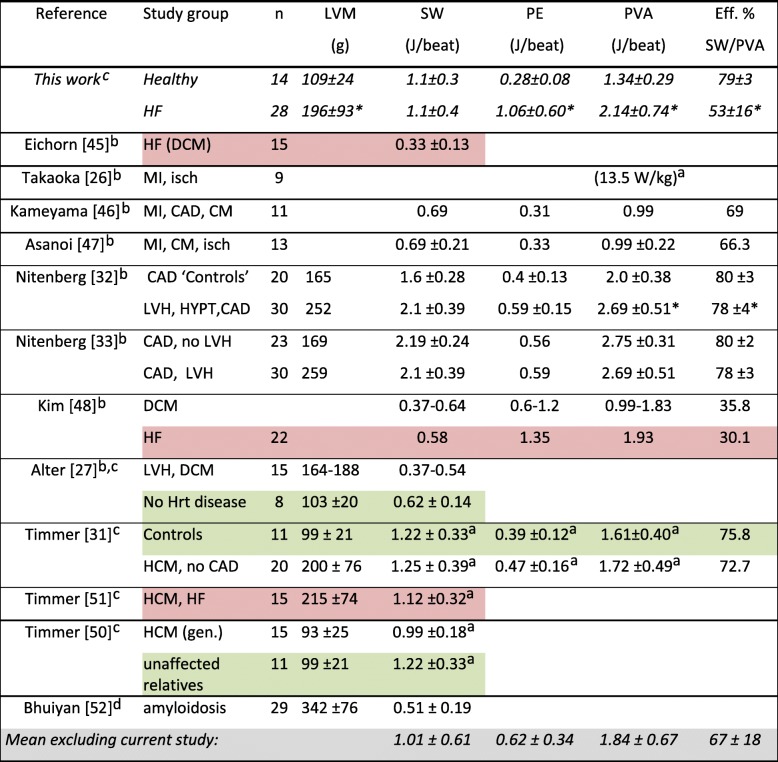
Comparison of noninvasive measurements of human cardiac mechanical work with prior studies employing catheterization techniques, ultrasound and cardiac MRI

*Eff* efficiency, *HF* congestive heart failure (prior studies, pink highlight), *DCM* dilated cardiomyopathy, *MI* prior myocardial infarction, *CAD* coronary artery disease, *CM* cardiomyopathy; *HYPT* hypertension, *LVH* left ventricular hypertrophy, *HCM* hypertrophic cardiomyopathy, *gen* genetic origin, *isch* patients with ischemia. Prior studies of patients who did not have heart disease are highlighted in green. *P < 0.05 vs. controls. ^a^Converted to SI units from mmHg.ml using a factor of 1.333 × 10^–4^. ^b^Used conductance and/or pressure catheter. ^c^Used cine CMR for volumetry. ^d^Used echocardiographic volumetry

While CMR is a gold standard for accurate measurements of myocardial volume, invasive intra-cardiac pressure monitoring throughout the cardiac cycle was impractical for our studies of subjects not undergoing catheterization. Consequently, obtaining the LV pressure from the brachial artery pressure is potentially confounding. A small systolic pressure difference between the brachial artery and the LV blood pool exists and is primarily related to central vascular stiffness. This can be indexed by the pulse-wave velocity, which in patients has been calibrated from catheter measurements to obtain the central aortic pressure [54]. Studies using this method to measure systolic aortic pressure in NYHA Class II-IV HF [53], hypertensives with coronary disease [54], and in another 10,613 subjects who were healthy, hypertensive or had heart disease [55], showed very similar aortic-peripheral artery pressure differences (healthy, 12 ± 6 mmHg, *n* = 5648; hypertensives, 12 ± 7 mmHg, *n* = 3420; cardiovascular disease, 12 ± 5 mmHg, *n* = 610) over a wide age range (18–101 years) [55]. As the aortic-brachial systolic pressure differential is the same or similar in patients and healthy subjects, it is unlikely to affect the significance of our findings other than by slightly decreasing the size of the CK energy reserve.

Computer simulations performed with stepped and half-sinusoid systolic pressure waveforms (see Additional file 1) suggest that p_p_ could underestimate the true peak mechanical power by up to  about 10%. This would again reduce the projected gap between CK energy supply and SW (the CK energy reserve), but should not affect the underlying conclusions. During diastole, simulations indicate that increasing end-diastolic pressure from 0 to 20 mmHg, alters p_av_ by only − 5% to + 3%, for P_sys_ ≥ 110 mmHg (per Table 1). Also as noted, the assumption of V_0_ = 0 has been used elsewhere [31]. It is supported by mean values of V_0_ = 0 ± 10, − 3 ± 11 ml, etc. measured using micromanometers and LV angiography during catheterization procedures performed on patients who had chest pain, some with hypertrophy [32, 33].

Patients in the present study had less severe HF and their reductions in cardiac CK rates and flux were less severe than those reported earlier [8, 17–19, 22]. Yet CK flux, which may be the most physiologically-relevant measure of CK metabolism for HF outcomes [18], was significantly reduced. There are presently no CK flux measurements available from HF patients during stress, but recent ^31^P MRS studies in hypertrophy [56] and diabetes [57] which are common precursors to HF, showed 8–12% decreases in cardiac PCr/ATP ratios during moderate exercise. Such reductions could further compromise CK energy supply during peak demand if they persisted in HF. Finally, the findings for this cohort of patients with non-ischemic cardiomyopathy may not be representative of all forms of HF. Reductions in cardiac CK kinetics *are* observed in nearly all experimental HF models from rodents to large animals and in common forms of human HF that arise from dilated, hypertrophic and ischemic cardiomyopathies. Whether the relationship between depressed cardiac CK energy supply and mechanical work seen here occurs in other forms of human HF remains to be seen.

## Conclusion

Whether the CK energy supply is much higher or lower than cardiac energy utilization bears directly on the CK shuttle hypothesis and the significance of the role played by cardiac CK energetics in the failing human heart. We measured both cardiac CK energy supply and mechanical work in healthy subjects and patients with HF for the first time. Using noninvasive CMR/MRS techniques, we found that reduced CK flux in HF at rest was associated with the same or similar reductions in peak and average stroke work. Although CK flux exceeded peak stroke work by almost 4-fold at rest, the loss in CK energy reserve combined with a reduction in mechanical efficiency in HF patients could limit energy supply during moderate activity or stress and mechanistically contribute to clinical HF events [18].

### Clinical implications

HF is a global pandemic affecting more than 20 million people worldwide. Despite advances in medical, device, and surgical therapies over the last three decades, the prognosis for patients with HF is still equivalent or worse than that of many cancers. New treatment strategies are needed. This work identifies inadequate energy supply as a potential contributor to the contractile dysfunction observed in patients with systolic HF which accounts for about half of the total HF population. The current findings are consistent with observations that medications which reduce energetic demand (eg, beta-blockers and/or angiotensin-renin blockade) significantly improve HF outcomes and survival. They also point to future ‘metabolic’ therapy strategies that could treat HF by restoring impaired energy supply, for example, by restoring mitochondrial function, augmenting the supply of alternate carbon substrates, or those that limit CK degradation [19] or genetically restore CK activity. Such energetic strategies may not necessarily replace, but could augment the efficacy of current HF treatment options.

## Additional file

Additional file 1: Supplementary material. (DOCX 79 kb)

## Abbreviations

ADP: Adenosine diphosphate
ATP: Adenosine triphosphate
BMI: Body mass index
BP: Blood pressure
CK: Creatine kinase
CMR: Cardiovascular magnetic resonance
EDV: End-diastolic volume
EF: Ejection fraction
ESV: End-systolic volume
HF: Heart failure
LV: Left ventricle/left ventricular
LVH: Left ventricular hypertrophy
LVM: Left ventricular mass
MRS: Magnetic resonance spectroscopy
ns: Not significant
NYHA: New York Heart Association
OXPHOS: Oxidative phosphorylation
PCr: Phosphocreatine
PE: Potential energy
PV: Pressure-volume
PVA: Total mechanical energy (or work)
SI: Système International
SW: Stroke work
