# Supplementary material for: Cardiac work is related to creatine kinase energy supply in human heart failure: a cardiovascular magnetic resonance spectroscopy study
Source: J Cardiovasc Magn Reson. 2018 Dec 10;20:81. doi: 10.1186/s12968-018-0491-6 (PMC6287363; doi:10.1186/s12968-018-0491-6)
Supplement: Supplementary file 1 — Supplementary material. (DOCX 79 kb) [file 12968_2018_491_MOESM1_ESM.docx]

# Additional file 1

# Cardiac Work is Related to Creatine Kinase Energy Supply in Human Heart Failure

# Refaat E. Gabr, AbdEl-Monem M. El-Sharkawy, Michael Schär, Gurusher S. Panjrath, Gary Gerstenblith, Robert G. Weiss, Paul A. Bottomley

## Protocol for measuring creatine kinase (CK) metabolites and energy supply

The magnetic resonance spectroscopy (MRS) studies were performed on a *Philips Healthcare* (Best, the Netherlands) 3 Tesla broadband *Achieva* magnetic resonance imaging (MRI) scanner using a 17-cm/8-cm phosphorus (^31^P) transmit/receive surface coil set with an embedded coil marker described previously[1]. Absolute concentrations of phosphocreatine, [PCr], and adenosine triphosphate, [ATP], were determined by ^31^P MRS using an external concentration referencing method that included corrections for coil loading, relaxation, heart motion, tissue volume, and coil sensitivity variations within voxels[2]. The CK reaction rate, *k_f_* was measured using triple repetition-time saturation-transfer (TRiST)[3]. The patient MRS protocol comprised the following steps (Fig. 1):

(1) Position the subject prone in the MRI scanner with the left ventricle above the ^31^P MRS coils, as confirmed by scout proton (^1^H) MRI.

(2) Shim the magnetic field homogeneity over the heart (water linewidth≈20 Hz)[4].

(3) Acquire coronal ^1^H MRI data for subsequent segmentation of cardiac tissue into MRS volume elements (voxels) for metabolite quantification.

(4) Switch to ^31^P MRS and acquire a fully-relaxed, cardiac-triggered (end-systole) one-dimensional (1D) spatially-localized, surface-coil detected chemical shift imaging (CSI) data from the chest and heart (16-phase-encodes; acquisitions per encode, NA=2; 1-cm resolution; repetition period TR≥15 s; echo time TE=1.4 ms; bandwidth=3 kHz) using adiabatic half-passage excitation (AHP; 5-ms tan/tanh-modulation; 7kHz frequency-sweep cycling). This acquisition is used to measure metabolite concentrations[1].

(5) Apply the ^31^P MRS TRiST method using three 1-cm resolution 1D CSI sequences applied with an amplitude-modulated frequency-selective saturation pulse train prior to AHP excitation[3,5]. The three sequences employ: (i) frequency-selective saturation of the γ-phosphate resonance of ATP (γ-ATP, -2.5 ppm relative to PCr) acquired at a TR of two heart-beats (~1.7 s; NA=18); (ii) the same saturation of the γ-ATP resonance but acquired with a cardiac-gated TR~10 s (NA=8); and (iii) frequency-selective saturation applied at +2.5 ppm acquired with a cardiac-gated TR~16 s (NA=2) as a control[6].

(6) To calibrate the MRS signal strength for measuring metabolite concentrations, the subject was replaced by a 6-cm diameter cylindrical concentration reference (30 mM NaH_2_PO_4_), and scanned with the same MRS protocol as Step 4 (but with TR=8 s). The three-dimensional (3D) spatial sensitivity profile of the ^31^P coil set was determined separately from a 3D CSI scan from a large phantom of 600 mM NaH_2_PO_4_ [2].

## MRS data analysis

MRS data were quantified to determine [PCr], [ATP] and *k_f_* from the corresponding MRS peak areas as detailed previously[2,3]. The ratio of the MRS signals from the coil marker measured in the spectra from the patient in Step 4 and from the reference phantom data from Step 6 were used to account for differences in MRS coil loading. Cardiac PCr was considered the primary endpoint for each MRS study because it is needed to determine [PCr], *k_f_,* and hence the CK flux which is given by the product, *k_f_*[PCr]. The signal-to-noise ratio (SNR) of PCr in cardiac spectra ranged from 8-40. Voxels with SNR <8 in spectra acquired for measuring [PCr] were generally not quantified because further reductions in SNR due to saturation transfer (Step 5i or 5ii) would the preclude *k_f_,* and CK flux measurements. The areas of the PCr, γ-ATP and blood 2,3-diphosphoglycerate (DPG) peaks in cardiac voxels in the data from Step 4, and that of the phosphate peak in the corresponding voxels from the concentration reference, were fitted using ‘Circle Fit’[7]. The ATP peak areas were corrected for blood ATP contamination by subtracting 15% of the DPG signal[8].

The volume of cardiac tissue contributing to each spectrum was determined by co-registering the cardiac MRIs from each subject with the reference phantom and the coil sensitivity profiles, using the embedded coil marker[2]. The myocardial tissue present in each MRS voxel was segmented by two different users (REG, PAB) who contoured the chamber walls in the coronal MRIs acquired in Step 3, and the two results averaged. The segmented tissue maps were multiplied by the coil sensitivity profile, and this process repeated for a segmented image of the concentration reference. The ratio of the resultant maps from the concentration reference and from the subject were used to provide a partial volume correction for each MRS voxel. The ratio of the metabolite peak areas to that of the reference in the same voxel was then multiplied by the partial volume correction factor and by the known reference concentration, to yield the metabolite concentration in mmol/liter of wet tissue[2]. This result was converted to mmol/kg wet weight using a cardiac specific gravity, SG=1.03[9].

The forward CK rate *k_f_* in sec^-1^, was determined from the steady-state PCr signal heights in the same cardiac voxels acquired in Step 5[3]. *k_f_* was calculated from:

| *k_f_ =* 0.0052(*M_0_`/M_0_*-0.8730)(*Q*+27.5332)(*T_1_`*-6.0647) | (A1) |
| --- | --- |

derived from a Bloch equation analysis that included the effects of spillover saturation[6,10]. Here, M_0_`, M_0_ and T_1_` are the PCr signals and the ^31^P MRS spin-lattice relaxation time of PCr obtained from the spectra acquired in Step 5(i-iii), and *Q* is the ratio of the PCr signal from Step 5(iii) to that from Step 4. The CK flux in mmol/(kg wt. sec) was calculated from the product, *k_f_*∙[PCr], and converted to *Système International* units of Watts/kg wet-tissue weight (W/kg) by multiplying by the free-energy of ATP hydrolysis, ΔG_ATP_=60 kJ/mol. The latter is the mean of 59-61 kJ/mol determined noninvasively in healthy and HF patients using the same methodology[11,12].

## Measuring cardiac stroke work

Cine MRI is a “gold standard” for noninvasive volumetry[13–15], and for pressure volume (PV)-loop measurements in large animals[16–21] and humans[19,21], wherein conductance catheters require volume calibration using an imaging modality. The PV-work, *w*(*t*), was evaluated from the pressure, *P(t),* and temporal (*t*) volume change, *∆V*, as:

|  | (A2) |
| --- | --- |

where the minus sign associates cardiac contraction with positive muscle work. The mechanical stroke work, SW, is the integral of Eq. (A2) over one cardiac cycle. The instantaneous mechanical power (or rate of energy consumption) is the derivative of Eq. (A2)[22]:

|  | (A3) |
| --- | --- |

Cine MRI and blood pressure (BP) measurements were performed with patients positioned either supine and scanned with 6- or 32-channel cardiac array coils, or prone using the scanner’s body MRI coil. These studies were performed as an add-on (Step 6) to the MRS protocol or as a separate exam, depending on patient preference and availability (Fig. 1). After shimming and performing scout MRI, double-oblique short-axis retrospective cardiac-gated MRI was performed in breath-held acquisitions using balanced steady-state free-precession (SSFP; 8-12 slices; TR =3.5 ms; echo time TE =1.8 ms; 30 cardiac phases; slice thickness =8 mm; slice gap =2mm; SENSE factor =2 for coil arrays; 1.6x1.3 mm in-plane scan resolution; 256x256 matrix; 1-2 slices/breath-hold; 15-25 min total MRI exam time). Systolic and diastolic BP were measured from an upper-arm cuff using an *In vivo Systems* (Orlando, FL) MRI-compatible pressure monitoring system before and after cine MRI, and averaged for PV-loop calculations.

Note that cardiac work is also converted to kinetic energy carried by the blood leaving the ventricles. Although the blood’s speed during contraction can reach ≈1m/s, the average is only ≈20cm/s[23]. For an average stroke volume of less than 100ml[24], this corresponds to only ≈2mJ which is minute (~0.2%) compared to the pump energy (≈1J), and was therefore neglected.

## MRI data analysis

The inner and outer contours of the left ventricle were manually delineated in all short-axis MRI sections at all time points. The left ventricular (LV) mass (LVM) was calculated from the difference between the two contours at end diastole summed over all slices and multiplied by SG. The LV blood volume was calculated at each cardiac phase by summing the blood volumes in all adjacent myocardial sections, and its derivative determined after temporal filtering to mitigate the effects of noise. The volume change was multiplied by the pressure waveform to calculate the rate of mechanical power consumption, per Eq. A3.

The stroke work (SW) was obtained from the integral of the PV work. The ‘potential energy’ (PE) was estimated from the line connecting the end-systolic PV-point in the PV-loops (Fig. 2), with an unstressed volume V_0_, assumed negligible[21]. The total mechanical energy (PVA) was estimated by the sum, SW+PE.

The effect of assuming a square pressure waveform was simulated by comparing a half-sinusoid pressure waveform during systole, starting and ending at the mean systolic and diastolic BP, respectively, as listed in Table 1 of the main manuscript. With a similar half-sinusoidal waveform for the volume, the square pressure waveform underestimated the peak power of the sinusoidal wave by 10.5%, and might thus be considered conservative. The effect on average power of neglecting diastolic pressure was investigated by linearly varying end-diastolic pressure (EDP) up to 20mmHg. The variations in EDP affected the average SW estimated from the square waveform model by -5.3% to 2.5% for P_sys_≥110mmHg (per Table 1).

***References***

1. El-Sharkawy A-M, Schär M, Ouwerkerk R, Weiss RG, Bottomley PA. Quantitative cardiac 31P spectroscopy at 3 Tesla using adiabatic pulses. Magn. Reson. Med. 2009;61:785–95.

2. El-Sharkawy A-MM, Gabr RE, Schär M, Weiss RG, Bottomley PA. Quantification of human high-energy phosphate metabolite concentrations at 3 T with partial volume and sensitivity corrections. NMR Biomed. 2013;26:1363–71.

3. Schär M, El-Sharkawy A-MM, Weiss RG, Bottomley PA. Triple repetition time saturation transfer (TRiST) 31P spectroscopy for measuring human creatine kinase reaction kinetics. Magn. Reson. Med. 2010;63:1493–501.

4. Schär M, Kozerke S, Fischer SE, Boesiger P. Cardiac SSFP imaging at 3 Tesla. Magn. Reson. Med. 2004;51:799–806.

5. Bodenhausen G, Freeman R, Morris GA. A simple pulse sequence for selective excitation in Fourier transform NMR. J. Magn. Reson. Elsevier; 1976;23:171–5.

6. Gabr RE, Weiss RG, Bottomley PA. Correcting reaction rates measured by saturation-transfer magnetic resonance spectroscopy. J. Magn. Reson. 2008;191:248–58.

7. Gabr RE, Ouwerkerk R, Bottomley PA. Quantifying in vivo MR spectra with circles. J. Magn. Reson. 2006;179:152–63.

8. Hardy CJ, Weiss RG, Bottomley PA, Gerstenblith G. Altered myocardial high-energy phosphate metabolites in patients with dilated cardiomyopathy. Am. Heart J. 1991;122:795–801.

9. Snyder WS. Report of the Task Group on Reference Man:... Prepared by a Task Group of Committee 2 of the International Commission on Radiological Protection: Adopted by the Commission in Oct. 1974. Pergamon; 1975.

10. Schär M, Gabr RE, El-Sharkawy A-MM, Steinberg A, Bottomley PA, Weiss RG. Two repetition time saturation transfer (TwiST) with spill-over correction to measure creatine kinase reaction rates in human hearts. J. Cardiovasc. Magn. Reson. Springer; 2015;17:1–11.

11. Weiss RG, Gerstenblith G, Bottomley PA. ATP flux through creatine kinase in the normal, stressed, and failing human heart. Proc. Natl. Acad. Sci. U. S. A. 2005;102:808–13.

12. Hirsch GA, Bottomley PA, Gerstenblith G, Weiss RG. Allopurinol Acutely Increases Adenosine Triphospate Energy Delivery in Failing Human Hearts. J. Am. Coll. Cardiol. 2012. p. 802–8.

13. Keller AM, Gopal AS, King DL. Left and right atrial volume by freehand three-dimensional echocardiography: in vivo validation using magnetic resonance imaging. Eur. J. Echocardiogr. 2000;1:55–65.

14. Rodevan O, Bjornerheim R, Ljosland M, Maehle J, Smith HJ, Ihlen H. Left atrial volumes assessed by three- and two-dimensional echocardiography compared to MRI estimates. Int. J. Card. Imaging. 1999;15:397–410.

15. Lang RM, Bierig M, Devereux RB, Flachskampf FA, Foster E, Pellikka PA, et al. Recommendations for chamber quantification: a report from the American Society of Echocardiography’s Guidelines and Standards Committee and the Chamber Quantification Writing Group, developed in conjunction with the European Association of Echocardiograph. J. Am. Soc. Echocardiogr. Mosby; 2005;18:1440–63.

16. Lin H-Y, Freed D, Lee TWR, Arora RC, Ali A, Almoustadi W, et al. Quantitative assessment of cardiac output and left ventricular function by noninvasive phase-contrast and cine MRI: validation study with invasive pressure-volume loop analysis in a swine model. J. Magn. Reson. Imaging. 2011;34:203–10.

17. Dori Y, Zviman M, Miller F, Halperin H, Rome JJ. New Approach to Pressure-Volume Loop Analysis Using Custom Made High Fidelity MRI Compatible Pressure Sensor Tipped Catheters and Real Time MRI. Circulation. AHA; 2011;124.

18. Witschey WR, Contijoch FJ, Pilla JJ, Dougherty L, Song HK, Levack MM, et al. Real time measurement of cardiac pressure-volume relationships. J. Cardiovasc. Magn. Reson. Springer; 2012;14:1–2.

19. Kuehne T, Yilmaz S, Steendijk P, Moore P, Groenink M, Saaed M, et al. Magnetic resonance imaging analysis of right ventricular pressure-volume loops: in vivo validation and clinical application in patients with pulmonary hypertension. Circulation. 2004;110:2010–6.

20. Russell K, Eriksen M, Aaberge L, Wilhelmsen N, Skulstad H, Remme EW, et al. A novel clinical method for quantification of regional left ventricular pressure-strain loop area: a non-invasive index of myocardial work. Eur. Heart J. 2012;33:724–33.

21. Schmitt B, Steendijk P, Lunze K, Ovroutski S, Falkenberg J, Rahmanzadeh P, et al. Integrated assessment of diastolic and systolic ventricular function using diagnostic cardiac magnetic resonance catheterization: validation in pigs and application in a clinical pilot study. JACC. Cardiovasc. Imaging. 2009;2:1271–81.

22. Westerhof N, Stergiopulos N, Noble MIMM. Snapshots of hemodynamics: an aid for clinical research and graduate education. Springer US; 2010.

23. Stalder AF, Russe MF, Frydrychowicz A, Bock J, Hennig J, Markl M. Quantitative 2D and 3D phase contrast MRI: optimized analysis of blood flow and vessel wall parameters. Magn. Reson. Med. 2008;60:1218–31.

24. Alfakih K, Plein S, Thiele H, Jones T, Ridgway JP, Sivananthan MU. Normal human left and right ventricular dimensions for MRI as assessed by turbo gradient echo and steady-state free precession imaging sequences. J. Magn. Reson. Imaging. 2003;17:323–9.
